# Bifunctional *Marsilea minuta* Biochar for Sustainable Hydrogen and Oxygen Production

**DOI:** 10.1021/acsomega.6c04638

**Published:** 2026-09-13

**Authors:** Marcos V. Q. Santos, Yslaine Andrade de Almeida, Cícero Inácio da Silva Filho, Lisiane dos Santos Freitas, Valter Doria Rocha Neto, Silvânio Silvério Lopes da Costa, Giancarlo R. Salazar-Banda, Eliana Midori Sussuchi

**Affiliations:** † Research Group in Electrochemical Sensors and nano(Materials) − SenM, Laboratory of Corrosion and Nanotechnology − LCNT, Graduate Program in Chemistry, Department of Chemistry, 74391 Federal University of Sergipe, São Cristóvão 49100-000, Brazil; ‡ Graduate Program in Chemistry, Department of Chemistry, 671396 Federal University of Sergipe, São Cristóvão 49100-000, Brazil; § Graduate Program in Engineering and Environmental Sciences, Federal University of Sergipe, São Cristóvão 49100-000, Brazil; ∥ Electrochemistry and Nanotechnology Laboratory, Institute of Technology and Research (ITP), Aracaju 49032-490, Brazil; ⊥ Graduate Program in Process Engineering (PEP), Tiradentes University, Aracaju 49032-490, Brazil

## Abstract

The high cost, scarcity, and limited stability of noble-metal-based electrocatalysts, such as platinum and rhodium, have motivated the search for more abundant, sustainable, and economically viable alternative materials for water electrolysis. In this context, biochar obtained from plant residues stands out as a promising carbonaceous material for the development of efficient electrocatalysts with low environmental impact. In this work, an electrocatalyst based on biochar derived from the aquatic plant *Marsilea minuta* was developed and evaluated for the hydrogen evolution reaction (HER) and oxygen evolution reaction (OER). Structural and chemical characterization revealed an amorphous, porous structure and the presence of oxygen- and nitrogen-containing functional groups, including carboxylic acids, and pyridinic N moieties, which may serve as active sites favorable for electrocatalytic reactions. Electrochemical analyses performed using a glassy carbon electrode modified with the material demonstrated significant activity toward both HER and OER, with low overpotentials of −0.19 and 0.36 V vs. the reversible hydrogen electrode (RHE), respectively, required to reach current densities of 10 mA cm^–2^, and Tafel slopes consistent with favorable reaction kinetics. Electrochemical stability was confirmed by chronopotentiometric measurements, highlighting the potential of the biochar (BMQ) as a low-cost, low environmental impact, bifunctional electrocatalyst.

## Graphical abstract

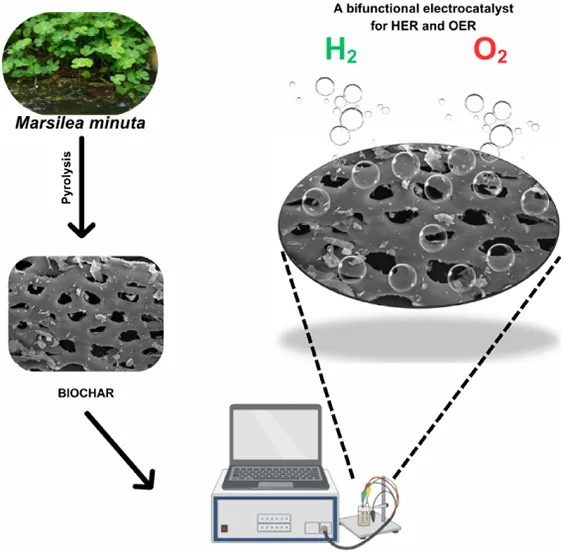

## 1 Introduction

The use of hydrogen as an energy storage vector has shown enormous potential across various sectors of modern society, including the aerospace industry, internal combustion engines, residential and industrial heating, among others.^1,2^ This is primarily because hydrogen combustion with oxygen provides a high gravimetric energy content relative to conventional fuels. Additionally, hydrogen has the lowest viscosity of all fluids, which can reduce friction and flow resistance, thereby minimizing efficiency losses in the form of dissipated heat.^3,4^

Currently, hydrogen production (90%–95%) is obtained from fossil fuel reforming, while only about 4% of global production comes from water electrolysis.^5^ Although hydrogen production from fossil fuels is a well-established and relatively low-cost technology, it still results in substantial greenhouse gas emissions and contributes to environmental pollution.^6^ Current hydrogen production technologies encompass a range of approaches, such as electrochemical water splitting, microbial and thermochemical conversions, solar-driven photoelectrochemical systems, and plasma-assisted processes, each presenting unique challenges and opportunities for sustainable energy development.^7^

Hydrogen production via water electrolysis is among the cleanest and most environmentally friendly methods available. However, current technologies still face a trade-off between cost and energy efficiency.^8^ In addition, water electrolysis commonly relies on electrocatalysts containing precious metals (e.g., platinum and iridium), which significantly increases the overall system cost.^6,9^ As an alternative to noble metal-based electrocatalysts, biochar, a solid product obtained through biomass pyrolysis and recognized as a potentially carbon-negative technology, can play an active or passive role in hydrogen production. In the active role, the material is used directly as a catalyst or catalyst support, whereas in the passive role, biochar is generated as a byproduct in processes such as biomass gasification and gas reforming.^10^

The hydrogen evolution reaction (HER) and oxygen evolution reaction (OER) are the key half-reactions in water electrolysis and are central to low-carbon hydrogen production.^11^ HER occurs at the cathode, involving the reduction of protons to hydrogen gas, while OER occurs at the anode through the oxidation of water and the release of molecular oxygen. Although conceptually straightforward, both reactions present kinetic challenges and require significant overpotentials, particularly OER. Therefore, the development of efficient, stable, and low-cost electrocatalysts is essential for large-scale, sustainable electrolysis.^12^

In this study, the aquatic species *Marsilea minuta* was used as a precursor for biochar production. At present, this plant has no major large-scale economic use, although it is cultivated as an ornamental species for aquariums. From an ecological standpoint, its choice as an alternative biomass is supported by its occurrence as an exotic species in various regions of Brazil, including sensitive areas such as the Caatinga. Due to its rapid growth and high adaptability, it may become invasive in aquatic ecosystems, competing with native species and affecting environmental balance.^13^ In this context, its use as a carbonaceous precursor may contribute to environmental management strategies while promoting the valorization of underutilized biomass resources. Furthermore, since it does not compete with food or established productive chains, it represents a low-impact material with potential for sustainable applications in electrocatalytic processes.

From a compositional perspective, *Marsilea minuta* is rich in flavonoids, alkaloids, phenolic compounds, and reducing sugars. It is recognized as an edible plant and shows antibacterial, cytotoxic, and antioxidant properties. More recently, it has also been investigated as a bioagent for the treatment of contaminated water.^14^ This phytochemical profile may favor the incorporation of heteroatoms such as nitrogen and oxygen into the carbon structure during pyrolysis, contributing to the formation of active sites in carbon-based materials.^15,16^ In addition, its fibrous structure may promote pore development during thermal treatment, resulting in materials with increased surface area and favorable ionic accessibility.^17^ For these reasons, *Marsilea minuta* biomass may exhibit favorable chemical reactivity and potential for electrochemical applications after thermal conversion.^18^ However, despite these promising features, to the best of our knowledge, no studies have reported the use of *Marsilea minuta*-derived biochar as a bifunctional electrocatalyst for HER and OER.

Thus, in this study, we produced and characterized a biochar derived from the exotic species *Marsilea minuta* and investigated its potential as a sustainable and low-cost electrocatalyst for HER and OER in water electrolysis systems. Given the importance of catalyst properties to electrolytic efficiency, special emphasis was placed on understanding how structural and chemical features of the biochar, such as porosity, surface roughness, and oxygen- and nitrogen-containing functional groups, affect its electrocatalytic behavior. By correlating these intrinsic material properties with the observed electrochemical performance, this study shows the feasibility of *Marsilea minuta* biochar as a promising alternative to conventional catalysts, contributing to the development of low-carbon technologies for hydrogen and oxygen production.

## 2 Experimental Section

### 2.1 Materials and Methods

Sulfuric acid (98.1%, H_2_SO_4_) was purchased from Quimex. Isopropyl alcohol (99.5%, C_3_H_8_O) and potassium chloride (99.5%, KCl) were obtained from Dinâmica. Potassium bromide (99.5%, KBr) was purchased from Merck. Ethanol (99.8%, C_2_H_5_OH) was obtained from Neon. Potassium hydroxide (99.0%, KOH) was obtained from Sigma-Aldrich. Nitrogen gas (N_2_, 5.0 grade) was supplied by White Martins. The glassy carbon electrode (MF-2052) was obtained from Bioanalytical Systems. All solutions were prepared using ultrapure water from a Milli-Q system (Millipore).

### 2.2 Preparation of *Marsilea minuta* Biochar

The biomass underwent pre-treatment as follows: *Marsilea minuta* was washed to remove impurities, dried at 80 °C for 24 h, and then ground using an industrial blender. Pyrolysis was conducted in a laboratory-scale stainless-steel pyrolyzer^19^ with an internal diameter of 5.30 cm and a length of 30.00 cm, externally heated by an electric furnace. In each run, 50 g of *Marsilea minuta* biomass was used. The temperature increased at a rate of 5 °C min^–1^ and was maintained for 1 h at 500 °C under a nitrogen atmosphere with a flow rate of 100.00 mL min^–1^. The resulting carbonaceous material was designated as biochar.

### 2.3 Physicochemical Characterization

Fourier transform infrared (FTIR) absorption spectra of the biomass and biochar samples were recorded using a PerkinElmer Spectrum Two FTIR spectrophotometer, with potassium bromide (KBr) employed as the dispersing agent. Surface elemental composition information was collected via X-ray photoelectron spectroscopy (XPS) using a Thermo Scientific K-Alpha spectrometer equipped with a standard monochromatic Al–Kα radiation source. XPS data processing and peak deconvolution were performed using CasaXPS software. Detailed fitting parameters are provided in Table S1. Thermogravimetric analysis (TGA) was performed to investigate the thermal stability and decomposition behavior of the biomass and biochar using a TA Instruments TGA 550. Raman spectra of the biochar were recorded using a HORIBA ExploraPlus instrument with a 532 nm diode laser and a beam power of 100 mW. Scanning electron microscopy (SEM) was used to investigate the morphological features of the biochar surface using a TESCAN VEGA 3 LMS SEM instrument operating at 15 kV, coupled with energy-dispersive X-ray spectroscopy (EDS) for elemental analysis. Phase identification of the materials was performed by powder X-ray diffraction (XRD) using a Rigaku RINT PC DMAX Ultima+ diffractometer with Cu Kα radiation (λ = 0.1540 nm) with a scan rate of 4° min^–1^. The point of zero charge (pHPZC) was determined by the 11-point method using aqueous solutions with initial pH values ranging from 2 to 12, followed by equilibration (24 h, 150 rpm) and final pH measurement. Nitrogen adsorption–desorption measurements were carried out at −196 °C using a Micromeritics ASAP 2020 surface area and porosity analyzer at the LABCAT—Laboratory of Catalysis and Materials, Department of General and Inorganic Chemistry, Institute of Chemistry, Federal University of Bahia (UFBA). Prior to the measurements, the biochar samples were degassed at 200 °C for 360 min under a nitrogen atmosphere to remove physically adsorbed species. The specific surface area was determined using the Brunauer–Emmett–Teller (BET) method. The total pore volume was estimated from the amount of nitrogen adsorbed at high relative pressure (P/P_0_ ≈ 0.99), while the average pore diameter was calculated from the adsorption and desorption branches of the isotherm. Micropore characteristics were evaluated using the Dubinin–Astakhov (DA) model, and the pore size distribution was determined using the Non-Local Density Functional Theory (NLDFT) model for slit-shaped carbon pores.

### 2.4 Electrochemical Measurements

Electrochemical analyses were performed using an Autolab PGSTAT M204 Potentiostat/Galvanostat controlled by NOVA 2.1.8 software in a conventional three-electrode configuration consisting of an Ag/AgCl (3.00 M KCl) reference electrode, a platinum wire (2 mm) counter electrode, and a glassy carbon working electrode (3 mm) in an electrochemical cell with a total volume of 70.00 mL. The electrolyte solutions were purged with N_2_ for 20 min. The current densities reported in this work were calculated by dividing the measured current by the geometric area of the working electrode. Potentials were converted to the reversible hydrogen electrode (RHE) scale using eq 1.

1 E(RHE)=E(Ag/AgCl)+0,197+0,0592xpH

The modified and unmodified glassy carbon electrodes (GCEs) were prepared and tested as follows. For the modified GCE, a suspension was prepared using 30.00 μL of perfluorinated Nafion resin (5 wt % in a mixture of lower aliphatic alcohols and water), 1.00 mL of absolute ethanol, and 0.0050 g of biochar (BMQ). The suspension was ultrasonicated for 40 min to ensure adequate dispersion. A 1.00 μL aliquot of the suspension was dropped onto the polished GCE surface and dried at room temperature. Linear sweep voltammetry (LSV) was used to determine the overpotential for HER in an acid medium (H_2_SO_4_ 2 M) and for OER in an alkaline medium (KOH 2 M). The scan rate was 1 mV s^–1^ for both HER and OER. All LSV analyses were preceded by an electrochemical activation step consisting of 100 cyclic voltammetry (CV) cycles; details are provided in the Supplementary Information (Figure S1), All electrochemical measurements were performed without iR compensation. The Tafel slope was obtained from the linear region of the Butler–Volmer relationship. Electrochemical impedance spectroscopy (EIS) measurements were recorded over the frequency range from 1 MHz to 1 Hz with a 10 mV amplitude at applied potentials of 0.06–0.40 V for HER and 1.30–1.60 V for OER, the equivalent circuit and the corresponding fitting parameters obtained for the HER and OER measurements are provided in the Supplementary Material (Figure S2 and Table S2). The electrochemical system was powered by photovoltaic energy. The electrochemically active surface area (ECSA) was calculated according to eq 2, assuming a specific capacitance (Cs) of 40 μF cm^–2^ for carbon-based materials.

2 $$ECSA=\left(\frac{Cdl}{Cs}\right)\times A_{geom}$$

To evaluate the bifunctional electrocatalytic activity, two-electrode measurements were carried out in a two-compartment H-cell separated by a Nafion 117 proton-exchange membrane. The cathodic compartment contained 2 M H_2_SO_4_, while the anodic compartment contained 2 M KOH, allowing the HER and OER to proceed under their respective optimized electrolyte environments. This experimental configuration consists of acid/alkaline water electrolysis systems, in which physically separated electrolytes enable different pH environments for each half-reaction. The solar power system consisted of a Jinko JKM565N-72HL4-V 565 W solar panel, a GROWATT OFF-GRID SPF5000ES 5 KVA 220 V inverter, and a MOURA 12MS234 stationary solar battery (12 V, 220 Ah).

## 3 Results and Discussion

### 3.1 Biochar Characterization

Several analyses were conducted to characterize the produced biochar. The thermal behavior of the biochar was evaluated by TGA, which provides information on the thermal stability of residual structural components and the carbonaceous matrix formed after pyrolysis. Thus, Figure 1A presents the thermogravimetric profile of the biochar sample, in which the black line corresponds to the mass-loss curve and the red line to its first derivative.

**Figure 1:**
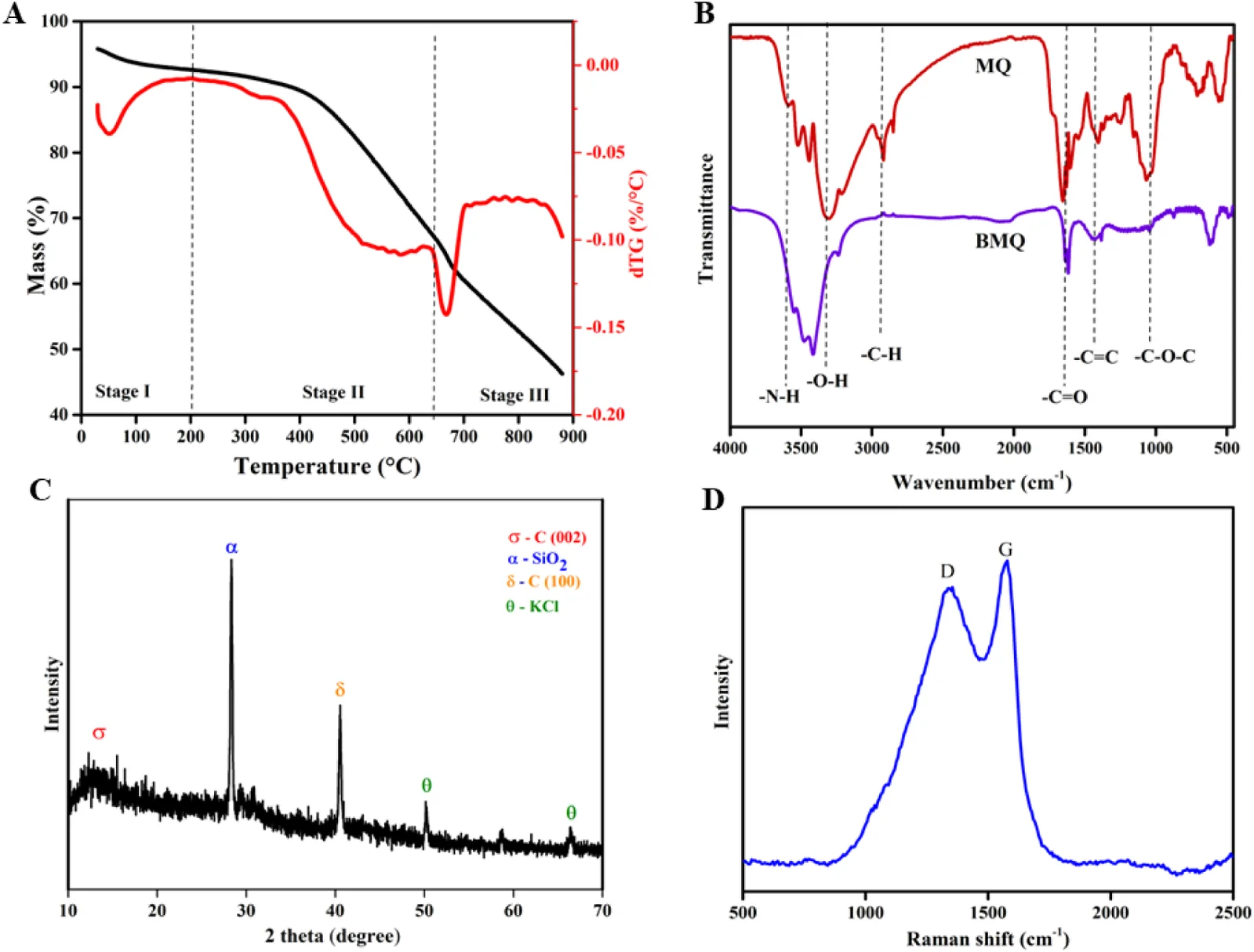
TGA curve and first derivative obtained for biochar sample under N_2_ atmosphere (A). Infrared absorption spectra of MQ and BIOCHAR in KBr, corresponding to biomass before pyrolysis and biochar, respectively (B). X-ray diffraction pattern obtained for the BIOCHAR (C). Raman spectrum of the biochar obtained from *Marsilea minuta* (D).

The mass-loss curve reveals three stages of thermal degradation. The first stage (25–150 °C) is attributed to the evaporation of water and low-molecular-weight volatile organic matter. Both the biomass and biochar samples exhibit a similar pattern during this stage. In the second stage, the biomass (Figure S3) shows a characteristic degradation event associated with the rapid decomposition of hemicellulose and cellulose, which begins at approximately 260 °C and reaches a maximum mass-loss at 320 °C due to the thermal cleavage of cellulose.^20^ These structural components of the biomass, such as cellulose and hemicellulose, are largely degraded during biochar production. Accordingly, in the second stage, the biochar exhibits greater thermal stability than the biomass, consistent with the formation of more thermally stable carbon forms.^20^ In the third stage, moderate mass loss is observed and is associated with lignin structures, which display higher thermal stability and are gradually decomposed over 300–900 °C.^21^ The thermogravimetric profile corroborates the FTIR results, which likewise indicate degradation of oxygen-containing functional groups and the formation of thermally stable compounds, as discussed below.

From Figure 1B, the biochar spectrum shows fewer absorption bands than the biomass, which is attributed to the degradation of surface functional groups during thermal treatment. Bands in the 3400–3300 cm^–1^ region are assigned to the stretching vibrations of hydrogen-bonded −OH groups (e.g., from polysaccharides, alcohols, and/or acids) and −NH_2_/NH–R groups (amines or amides). The band at 2920 cm^–1^ is related to the symmetric stretching of C–H bonds in aliphatic groups. Bands between 1610 cm^–1^ and 1350 cm^–1^ correspond to stretching vibrations of C=C and C=O bonds.^22–24^ The decrease in C–O–C-related bands is consistent with cleavage of ether linkages during pyrolysis.^25^ These spectral changes are consistent with lignocellulosic materials subjected to pyrolysis, indicating conversion of the biomass into a more stable carbonaceous material with lower oxygen functionality and a higher degree of aromaticity.

The physical nature of the biochar sample was evaluated by XRD analysis. The XRD pattern (Figure 1C) reveals the presence of both crystalline and amorphous components. The broad, low-intensity diffraction halo shown in Figure 1C is characteristic of amorphous or partially graphitic materials. During biochar production, crystalline cellulose is degraded, resulting in a disordered carbonaceous structure. This feature may be advantageous for electrocatalytic applications because it can increase the exposure of accessible active sites and facilitate charge transport.^17,26^ A second feature in the 42–46° region corresponds to the C(100) plane, indicating the presence of condensed aromatic carbon structures. The appearance of these peaks suggests some degree of structural ordering within the carbon matrix, although the material remains predominantly disordered.^27^

The peaks at specific 2θ values were assigned to crystalline phases attributed to silicon dioxide (SiO_2_) at 28.30° and potassium chloride (KCl) at 50.10° and 66.44°, according to JPCDS cards 46-1045 (SiO_2_) and 41-1476 (KCl). The presence of SiO_2_ may indicate residual minerals from the original biomass, as amorphous silicates can crystallize during pyrolysis.^28^ The KCl peaks are likely associated with naturally occurring minerals in the precursor biomass that remain as inorganic constituents after pyrolysis.^27^ These dispersed crystalline phases may influence electrochemical behavior; however, direct evidence for a catalytic role or conductivity enhancement is not provided here.

The degree of graphitization of the biochar was estimated by Raman spectroscopy (Figure 1D). The spectrum displays two distinct peaks at approximately 1355 and 1578 cm^–1^, corresponding to the D and G bands, respectively. The D band is associated with disordered graphitic domains and is assigned to the A_1_g vibrational mode of the carbon lattice, whereas the G-band is attributed to the E_2_g symmetry of the in-plane stretching mode of sp^2^-hybridized carbon in layered graphitic structures.^26,29^ The fitted intensity ratio between the D and the G-bands was 0.91, indicating a partially graphitic structure with a moderate degree of disorder. This structural configuration may be beneficial for electrochemical applications because surface defects can increase the density of accessible active sites, while graphitic domains can help maintain electrical conductivity for charge transfer, thereby facilitating the desired electrochemical reactions.

XPS analysis of the biochar, shown in Figure 2, revealed that the surface is predominantly composed of carbon, oxygen, and nitrogen. As presented in Figure 2A, the survey spectrum displayed characteristic binding energy peaks corresponding to O 1s (531.38 eV), C 1s (284.17 eV), and N 1s (398.99 eV), confirming the presence of these elements on the material surface.^30^

**Figure 2:**
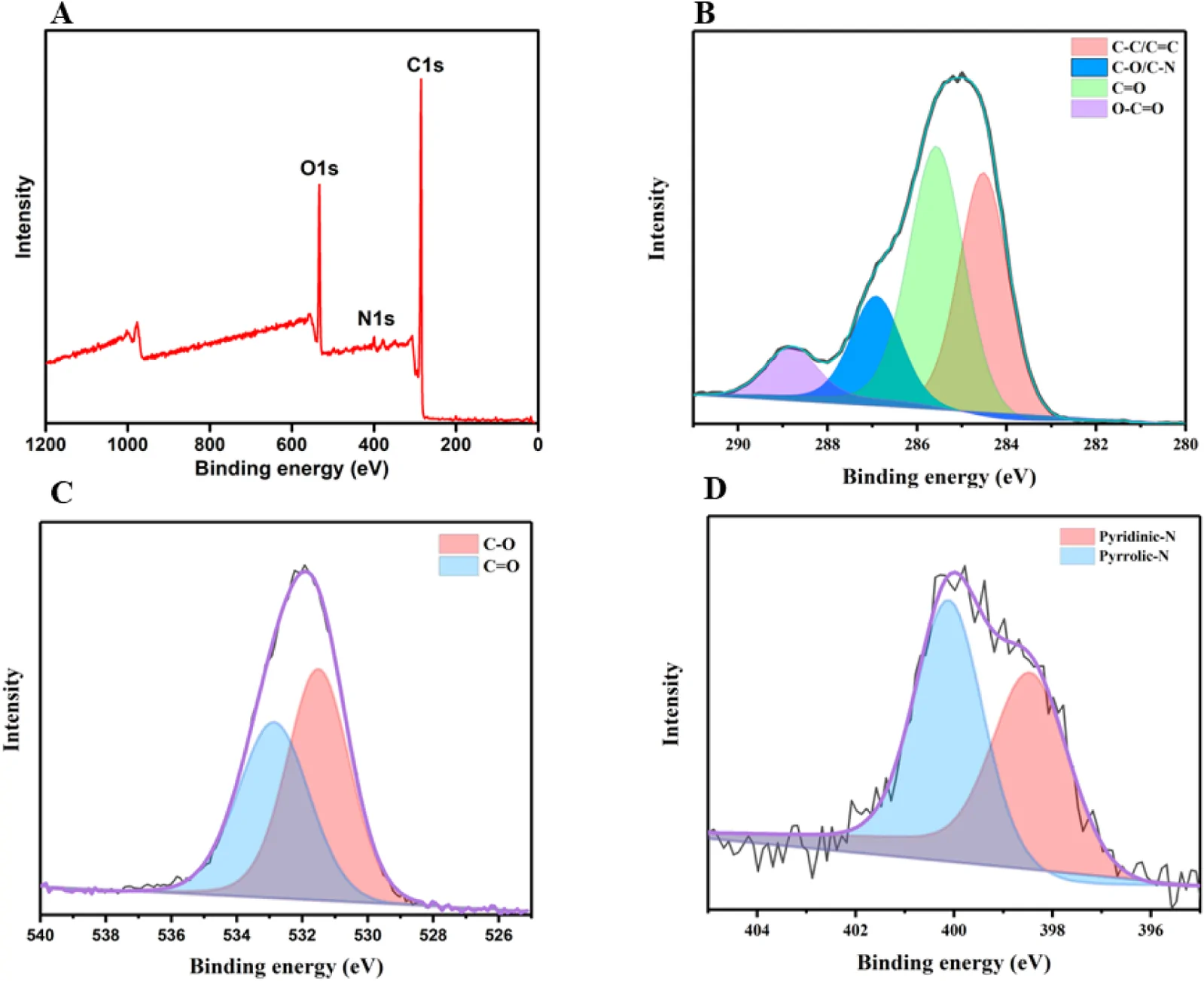
XPS survey spectrum of BMQ (A). High-resolution XPS spectra of the C 1s region (B), O 1s region (C), and N 1s region (D).

Figure 2B shows the deconvolution of the C 1s peak, with four main components at 284.50, 285.55, 286.93, and 288.80 eV, corresponding to C–C/C=C (sp^2^ carbon), C–O/C–N, C=O, and carboxylic groups (O–C=O), respectively. These results suggest a surface functionalized with oxygen- and nitrogen-containing organic groups.^31^ In Figure 2C, the O 1s spectrum shows two contributions at 531.33 and 533.00 eV, associated with C=O-containing species and O–C=O/C–O groups, respectively, supporting the presence of oxygenated surface functionalities. The N 1s spectrum (Figure 2D) confirms the presence of nitrogen-containing species. The peaks at 398.44 and 400.09 eV are attributed to pyridinic nitrogen and pyrrolic nitrogen, respectively. These surface species may contribute to surface basicity and adsorption properties.^32^

The data in Table S1 indicate that most of the carbon detected by XPS in biochar is associated with oxygen-containing groups, mainly C–O/C–N (44.86%), followed by graphene-type bonds (C–C/C=C, 33.74%), carbonyl groups (C=O, 13.94%), and carboxylic groups (O–C=O, 7.46%). Analysis of the N 1s region showed that nitrogen is present mainly in the pyrrolic form (51.89%), with a similar contribution from pyridinic nitrogen (48.11%). These functionalities may provide chemically active surface sites relevant to adsorption and heterogeneous catalysis.^33^ The elemental composition and surface functionalities were also evaluated by complementary techniques, including EDS (Figure S4 and Table S3), which consistently confirmed the predominance of C, O, and N species.

According to previous theoretical and experimental studies, oxygen- and nitrogen-containing functionalities can regulate the local electronic structure of carbon materials by redistributing the electron density around neighboring carbon atoms.^34^ Because oxygen and nitrogen possess higher electronegativities than carbon, their incorporation into the carbon framework induces local electronic polarization, generating carbon sites with modified electron density that are more favorable for the adsorption of reaction intermediates and interfacial electron-transfer processes.^35^ Pyridinic nitrogen has been identified as one of the most influential nitrogen configurations in heteroatom-doped carbon materials. Combined experimental and theoretical studies have demonstrated that pyridinic N modifies the electronic structure of the adjacent carbon atoms, increasing their Lewis basicity and making these neighboring carbon atoms, rather than the nitrogen atoms themselves, the preferred adsorption sites for reaction intermediates.^36^

The point of zero charge (PCZ) of the biochar was determined by the 11-point method, resulting in a value of 6.25 (Figure S5). This value shows that, at pHs below 6.25, the biochar surface tends to exhibit a net positive charge due to the protonation of surface functional groups, especially oxygenated (such as phenolic and carboxylic) and nitrogenated (such as pyridine) groups. This protonation reinforces the positive character of the surface in acidic media. At pH values above the PCZ, these groups tend to deprotonate, leading to a net negative surface charge.^37^ This characteristic may be relevant for electrocatalytic reactions; high surface proton (H^+^) coverage can reduce the energy barrier associated with the hydrogen adsorption (H_ad_) step, resulting in lower energy expenditure for the initiation of the reaction.

SEM images (Figure 3) revealed the heterogeneous surface morphology of the obtained biochar, with irregular features and the presence of macro- and microporous regions. The porous, honeycomb-like structure is characteristic of thermally treated plant biomass and suggests partial preservation of natural anatomical channels even after pyrolysis.^33^ The surface also exhibited a rough texture, which may affect gas bubble nucleation, growth, and detachment during gas-evolving reactions (e.g., HER and OER), potentially influencing apparent activity and stability.^37^

**Figure 3:**
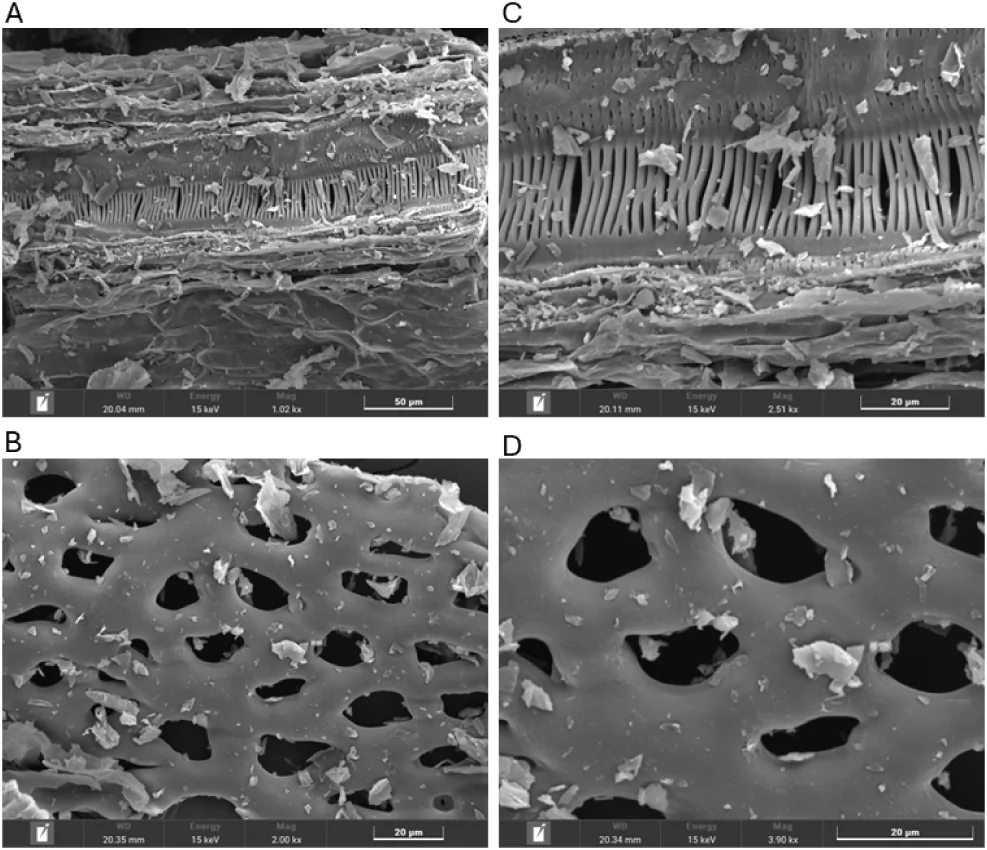
SEM images of BMQ obtained taken at different magnifications: 1.02 k× (A), 2.51 k× (B), 2 k× (C), and 3.9 k× (D).

To quantitatively evaluate the textural properties suggested by the SEM observations, nitrogen adsorption–desorption measurements were performed. The biochar exhibited a BET specific surface area of 0.95 m^2^ g^–1^, a total pore volume of 0.00267 cm^3^ g^–1^, and average pore diameters of 11.21 nm (adsorption) and 9.71 nm (desorption), indicating limited nitrogen-accessible porosity. The relatively low BET surface area may be attributed to the complex nature of biomass-derived carbons, whose textural properties are governed by multiple factors beyond the pyrolysis temperature alone. Previous studies have demonstrated that the final surface area and pore structure of biochars are strongly influenced by the biomass precursor, heating rate, residence time at the final pyrolysis temperature, particle size, and the intrinsic composition of the feedstock.

### 3.2 HER Performance Evaluation

LSV was used to investigate the electrocatalytic activity of the synthesized material toward HER (Figure 4A). The bare GCE exhibited no significant HER electrochemical response, whereas the biochar-modified electrode demonstrated remarkable electrocatalytic activity, requiring a potential of only −0.19 V to reach a current density of 10 mA cm^–2^. This current density is commonly used as a benchmark for evaluating the efficiency of photoelectrochemical water-splitting systems, making it a reference point for comparing different electrocatalysts. The observed activity may be related to oxygen- and nitrogen-containing surface groups, as evidenced by FTIR and XPS. These functionalities may act as active sites and contribute to interfacial charge transfer and hydrogen adsorption/desorption processes.^38,39^

**Figure 4:**
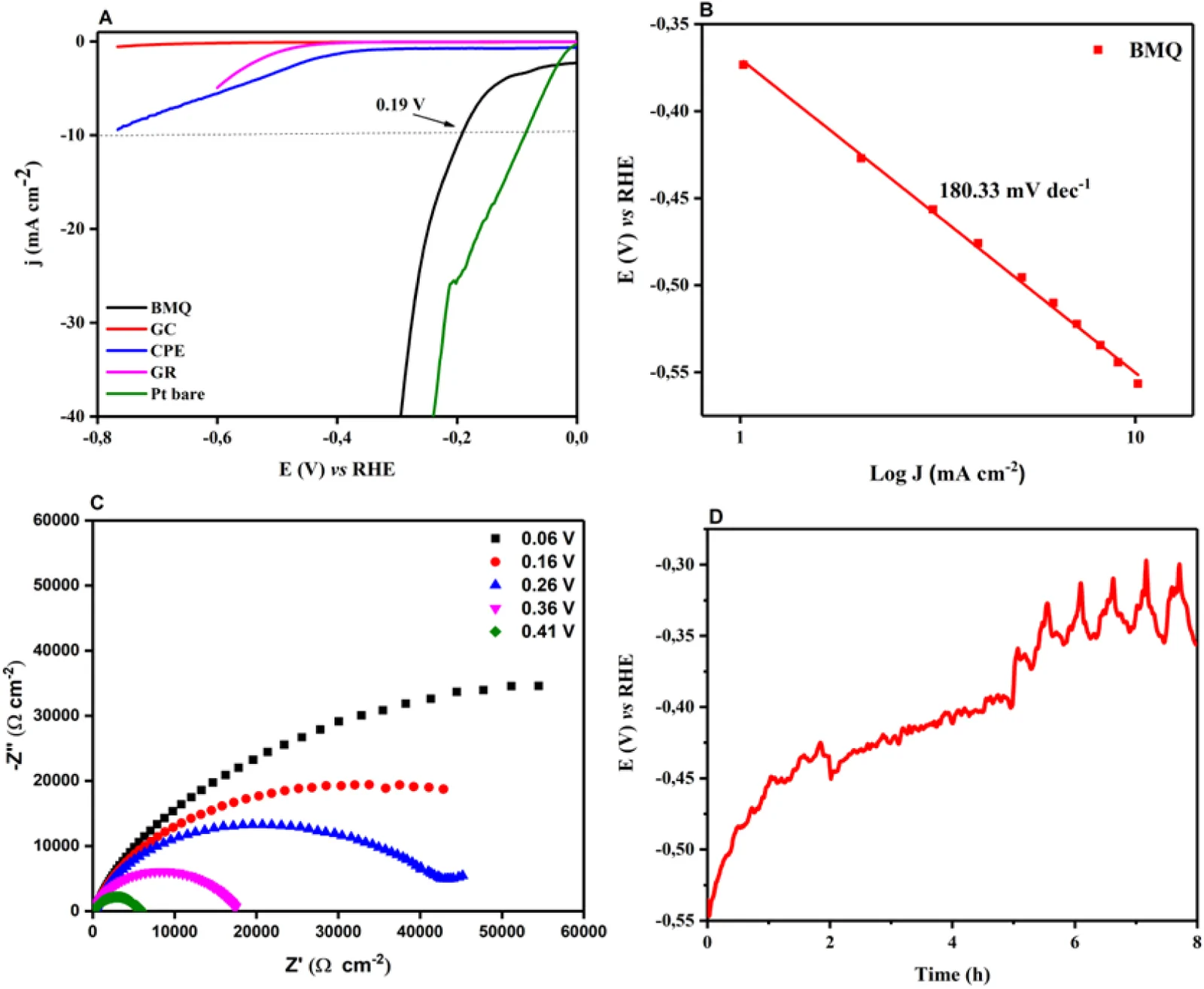
(A) Linear sweep voltammograms for HER using biochar modified electrode (BMQ), Glassy carbon electrode (GC) and Pt bare electrode (Pt); (B) Tafel plot of the BMQ sample; (C) Nyquist plots recorded at different potentials; and (D) Chronopotentiometric stability test under a current density of 10 mA cm^–2^ using biochar modified electrode (BMQ).

The Tafel slope obtained for the *Marsilea minuta*-derived biochar (Figure 4B) was 180.33 mV dec^–1^, indicating sluggish apparent HER kinetics under the investigated conditions. Although Tafel slopes are often used to infer reaction mechanisms, recent studies have shown that the measured values may deviate substantially from classical theoretical limits due to catalyst-layer effects, surface heterogeneity, and local mass-transport phenomena. Therefore, the observed value should not be interpreted as direct evidence of a specific rate-determining step.^40^

The elevated apparent Tafel slope may arise from a combination of factors. In addition to possible local H_2_ transport limitations within the porous biochar layer, the chemically heterogeneous distribution of oxygen- and nitrogen-containing surface functionalities can lead to non-uniform hydrogen adsorption energies and interfacial charge-transfer behavior.^41^ Moreover, based on the experimentally determined point of zero charge, the acidic electrolyte used in the HER measurements places the biochar surface below its PCZ, favoring a positively charged/protonated surface state. This condition may influence the adsorption–desorption equilibrium of HER intermediates and contribute to the non-ideal apparent kinetics. Consequently, the measured slope likely reflects the combined influence of intrinsic reaction kinetics, interfacial charge effects, and local transport limitations associated with the porous and chemical heterogeneous nature of the biochar.^41,42^

EIS was employed to evaluate the interfacial response of the BMQ biochar at different applied potentials near the HER potential region. The Nyquist plots (Figure 4C) show a decrease in the semicircle diameter with increasing cathodic polarization, which is consistent with a reduction in charge-transfer resistance (R_ct_) and faster interfacial electron-transfer kinetics.^43^ To quantitatively support this interpretation, the EIS spectra were fitted using the equivalent circuit *R_s_* – (*R*_*ct*_ ∥ CPE), and the corresponding fitting parameters are provided in Table S2. In addition, heteroatoms (e.g., N, S, P, and O) can contribute to non-ideal capacitive behavior and possible pseudocapacitive responses. Such pseudocapacitance can arise from surface redox processes and/or specific ion adsorption accompanied by electron transfer at redox-active sites.^44–46^

The double-layer capacitance (Cdl) of BMQ was determined by cyclic voltammetry (Figure S6). The Cdl value for BMQ was 164.07 μF cm^–2^, confirming the presence of a rough and electrochemically accessible porous surface with active sites exposed to the electrolyte. Assuming a specific capacitance (Cs) of 40 μF cm^–2^ for a flat carbon surface, a roughness factor (RF = Cdl/Cs) of 4.10 was obtained, indicating that the electrochemically accessible area is approximately four times larger than the geometric area of the electrode. Based on the geometric area of the glass carbon electrode (0.07 cm^2^), the estimated ECSA was 0.287 cm^2^. However, this value should be regarded as an approximation because Cs may vary depending on the surface chemistry and pore structure of carbonaceous materials.^47,47–49^

The chemical and mechanical stability of the material was evaluated using chronopotentiometry. The chronopotentiometric profile shown in Figure 4D displays three stages. In the first stage (0–2 h), the potential was approximately −0.55 V and drifted toward −0.45 V, corroborating electrode activation already observed in cyclic voltammetry. In the second stage (2–4 h), the potential remained relatively stable with minor fluctuations, indicating that the electrocatalyst reached electrochemical equilibrium, operating continuously and stably for hydrogen evolution. From the fifth hour onward (third stage), periodic oscillations in the potential signal were observed, with an average value near −0.05 V. This pattern is consistent with the formation and release of hydrogen bubbles on the electrode surface, as discussed earlier.^49^

### 3.3 OER Evaluation Performance

The LSV curves shown in Figure 5A indicate that BMQ reached a current density of 10 mA cm^–2^ at a potential of approximately 1.56 V. This behavior consists of an active role of the biochar in accelerating the OER kinetics, potentially associated with oxygen- and nitrogen-containing surface functionalities (as indicated by XPS) and the highly accessible surface structure that can facilitate interfacial charge transfer.^50,51^

**Figure 5:**
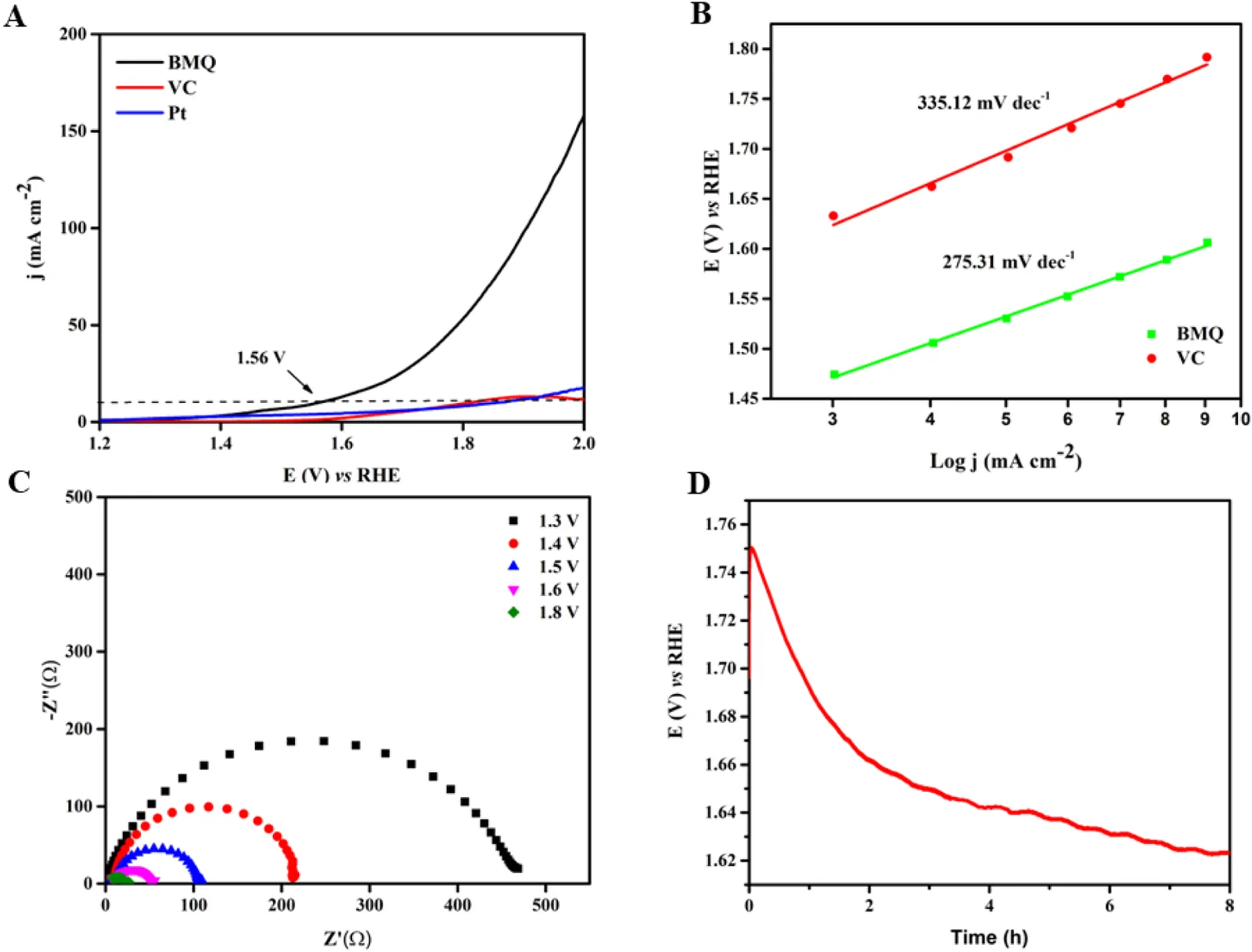
(A) Linear sweep voltammograms for OER using biochar modified electrode (BMQ), Glassy carbon electrode (GC) and Pt bare electrode (Pt). (B) Tafel plots for the BMQ sample; (C) Nyquist plots recorded at different potentials; (D) Stability test under a current density of 10 mA cm^–2^ using biochar modified electrode (BMQ).

The Tafel analysis presented in Figure 5B shows a significant improvement in the performance of the electrode containing biochar. This electrode showed a lower Tafel slope (273.31 mV dec^–1^) than the unmodified electrode (335.12 mV dec^–1^), consistent with comparatively more favorable OER kinetics under the evaluated conditions. Given the relatively large Tafel slopes in both cases, the improvement is interpreted as a reduction in overall polarization (including ohmic, charge-transfer, and mass-transport contributions) rather than definitive evidence of a change in the intrinsic activation-energy barrier. Consistent with this trend, EIS (Figure 5C) shows that R_ct_ decreased as the applied potential increased, indicating faster interfacial charge transfer at more anodic polarization.^52^ To quantitatively support this interpretation, the EIS spectra were fitted using the equivalent circuit *R_s_* – (*R*_*ct*_ ∥ CPE), and the corresponding fitting parameters are provided in Table S2. The lowest resistance was observed near the OER onset region identified by LSV (approximately 1.60 V), in agreement with the polarization results.

The stability profile (Figure 5D) shows a decrease in the potential required to sustain 10 mA cm^–2^, starting at approximately 1.75 V vs. RHE and gradually decreasing until stabilizing around 1.62 V after approximately 7 h. This decrease in potential overtime may be attributed to progressive activation of the electrocatalytic surface, where sustained operation can promote surface wetting, restructuring, and/or exposure of additional active sites.^53^ The absence of abrupt oscillations in the monitored potential during the test suggests stable operation within the monitored time window, and the decrease in potential indicates a lower energy requirement to maintain the target current density during continuous operation.

The bifunctional activity of BMQ was confirmed by replacing the bare glassy carbon anode with a BMQ-modified anode in a two-electrode configuration. As shown in Figure S7, only a small potential shift of approximately 10 mV was observed at a current density of 100 mA cm^–2^. This minor increase in polarization indicates that BMQ remains electrochemically active when used as both cathode and anode, demonstrating its ability to promote both half-reactions and supporting its bifunctional electrocatalytic behavior. To provide a broader context, Table S4 compares the electrocatalytic performance of BMQ with previously reported catalysts. The long-term stability of the catalyst was evaluated by chronopotentiometry at a constant current density of 10 mA cm^–2^ for 25 h, as shown in Figure 6.

**Figure 6:**
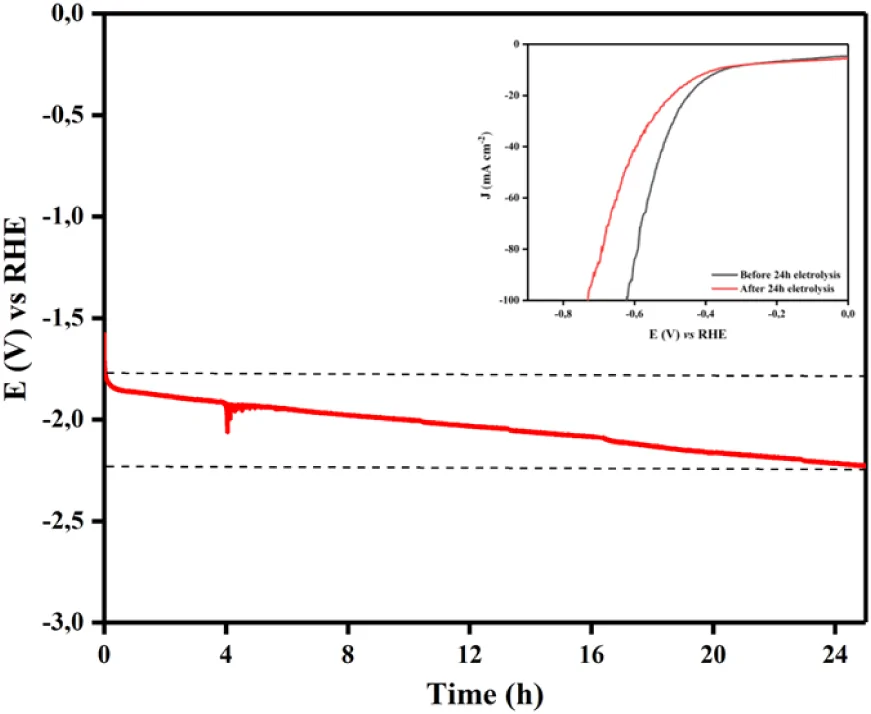
Chronopotentiometric stability test of the BMQ-modified electrode at 10 mA cm^–2^ for 25 h in 2.0 mol L^–1^ H_2_SO_4_. Inset: comparison of the HER polarization curves before and after the stability test.

The observed performance decay is likely associated with the combined effects of the electrode architecture rather than solely with intrinsic catalyst degradation. Since the catalyst layer was prepared using Nafion-containing ink deposited onto a glassy carbon electrode, partial coverage of active sites by the ionomer and changes in its hydration state may decrease catalyst utilization during prolonged electrolysis. Moreover, the continuous nucleation, growth, and detachment of hydrogen bubbles can generate mechanical stress on the catalyst film, promoting partial catalyst detachment from the substrate and reducing the number of accessible active sites.^54,55^ These effects are consistent with recent studies highlighting the influence of polymer binders, catalyst layer configuration, and bubble dynamics on the long-term performance of gas-evolving electrocatalysts.^56,57^

The compiled data indicates that BMQ exhibits competitive overpotentials and Tafel slopes for both HER and OER, including relative to some metal-doped or heteroatom-enriched biochar materials. These findings support *Marsilea minuta*-derived biochar as a bifunctional electrode material under the tested HER (acidic) and OER (alkaline) conditions.

## 4 Conclusions

In this study, a biochar derived from *Marsilea minuta* (an exotic plant species in Brazil) was produced by direct pyrolysis of the plant precursor. The resulting material exhibited a porous structure and rough surface features and contained oxygenated functionalities (e.g., carboxyl groups) and pyrrolic/pyridinic nitrogen. These surface functionalities can modify the electronic structure of the carbon matrix and provide adsorption sites that may participate in HER and OER. Electrochemical analyses showed that the biochar-modified electrode was highly active for both HER and OER, reaching 10 mA cm^–2^ with competitive overpotentials of 0.19 V (HER) and 0.36 V (OER). The Tafel slope for HER (180.33 mV·dec^–1^) indicates kinetically limited behavior under the tested conditions and is consistent with adsorption-influenced kinetics on a heterogeneous surface rather than supporting a single dominant elementary step. For OER, the biochar-modified electrode exhibited a lower Tafel slope than the unmodified electrode, consistent with comparatively more favorable apparent OER kinetics under the evaluated conditions. Overall, these results support the feasibility of *Marsilea minuta*-derived biochar as a sustainable, low-cost bifunctional electrode material for alkaline water electrolysis.

## Supplementary Material



## Notes

Additional electrochemical characterization data (CV activation curves, scan rate analysis, and pH variation studies), thermal analysis (TGA and derivative curves), surface and elemental characterization (XPS and EDS data with corresponding tables), and a comparative table of electrocatalytic performance for HER and OER in alkaline media, along with the associated references (PDF)
