# Supplementary material for: Bifunctional Marsilea minuta Biochar for Sustainable Hydrogen and Oxygen Production
Source: ACS Omega. 2026 Sep 13;11(38):57153–63. doi: 10.1021/acsomega.6c04638 (PMC13628611; doi:10.1021/acsomega.6c04638)
Supplement: Supplementary file 1 [file ao6c04638_si_001.pdf]

Supporting Information

Bifunctional *Marsilea minuta* Biochar for Sustainable Hydrogen and Oxygen Production

Marcos V. Q. Santos[a], Yslaine Andrade de Almeida[b, d], Cícero Inácio da Silva Filho[a], Lisiane dos Santos Freitas[b], Valter Doria Rocha Neto[b], Silvânio Silvério Lopes da Costa[c], Giancarlo R. Salazar-Banda[d,e], Eliana Midori Sussuchi[a*]

[a] Research Group in Electrochemical Sensors and nano(Materials) - SEnM, Laboratory of Corrosion and Nanotechnology - LCNT, Graduate Program in Chemistry, Department of Chemistry, Federal University of Sergipe.

[b] Graduate Program in Chemistry, Department of Chemistry, Federal University of Sergipe.

[c] Graduate Program in Engineering and Environmental Sciences, Federal University of Sergipe.

[d] Electrochemistry and Nanotechnology Laboratory, Institute of Technology and Research (ITP), 49032-490, Aracaju, Brazil.

[e] Graduate Program in Process Engineering (PEP), Tiradentes University, 49032-490, Aracaju, Brazil.

Corresponding author, E-mail: esmidori@gmail.com

Content

Figures..3

Tables...6

References...10

FIGURES

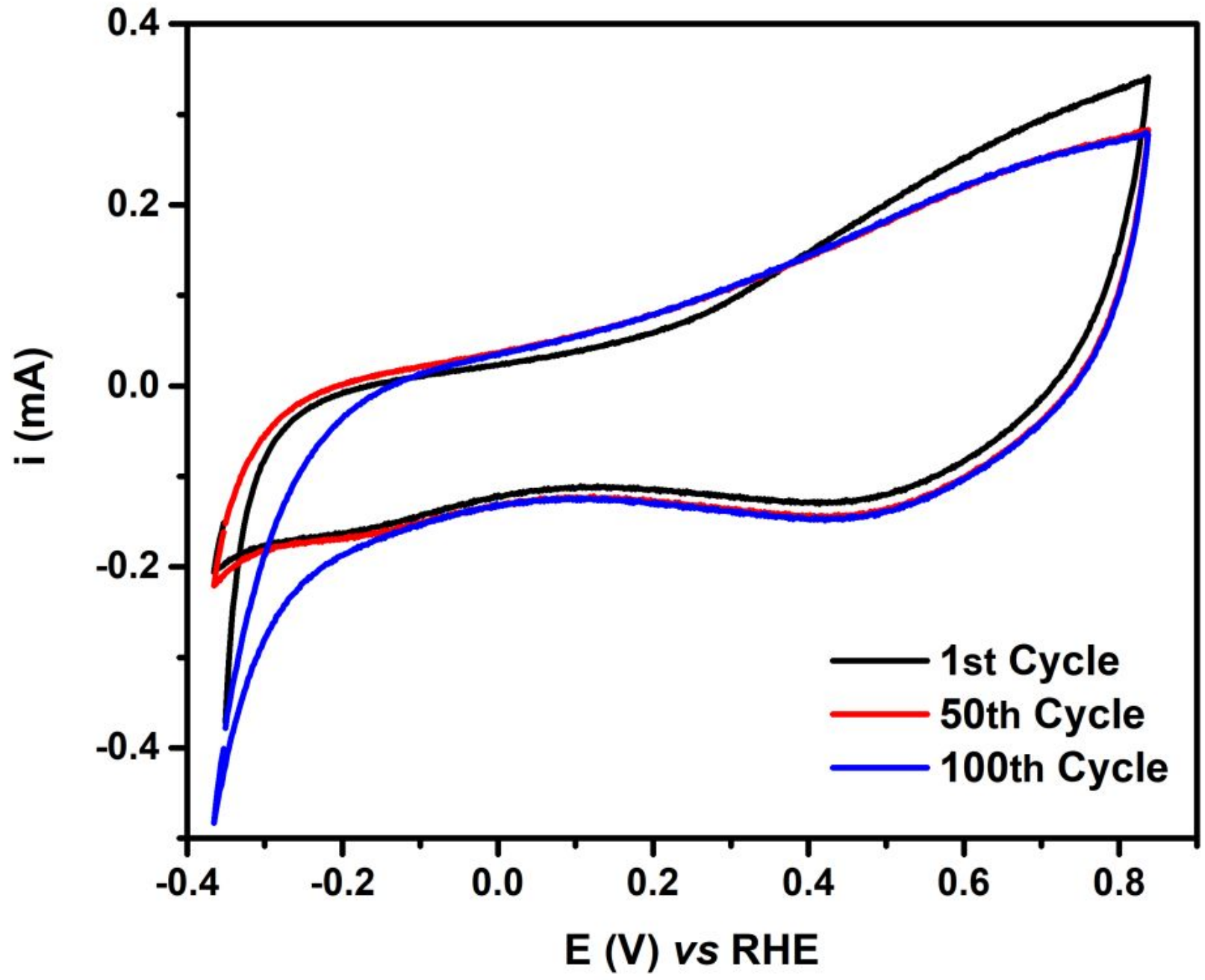

Fig. S1 Cyclic voltammetry (CV) activation of the electrode prior to LSV measurements, recorded in 2.0 mol L^{-1} H_2SO_4 at a scan rate of 100 mV s^{-1}. Profiles correspond to the 1st, 50th, and 100th cycles.

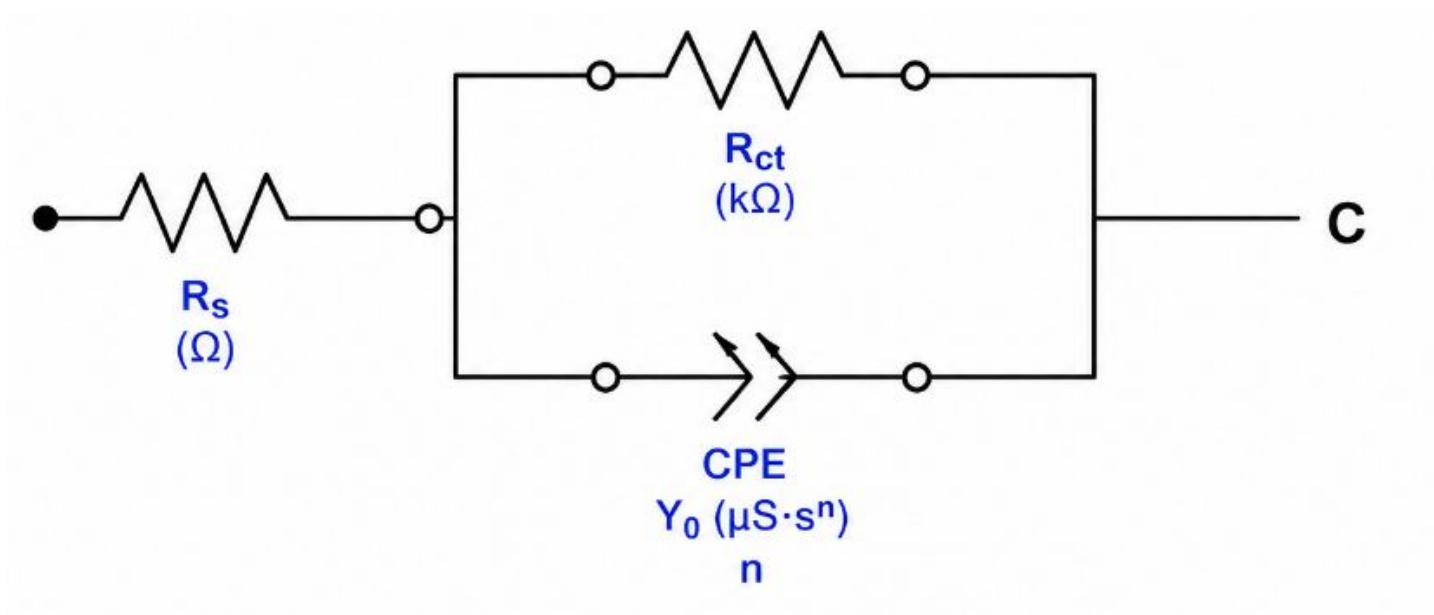

Figure S2 Equivalent circuit model used to fit the EIS spectra collected at different applied potentials for the HER and OER measurements.

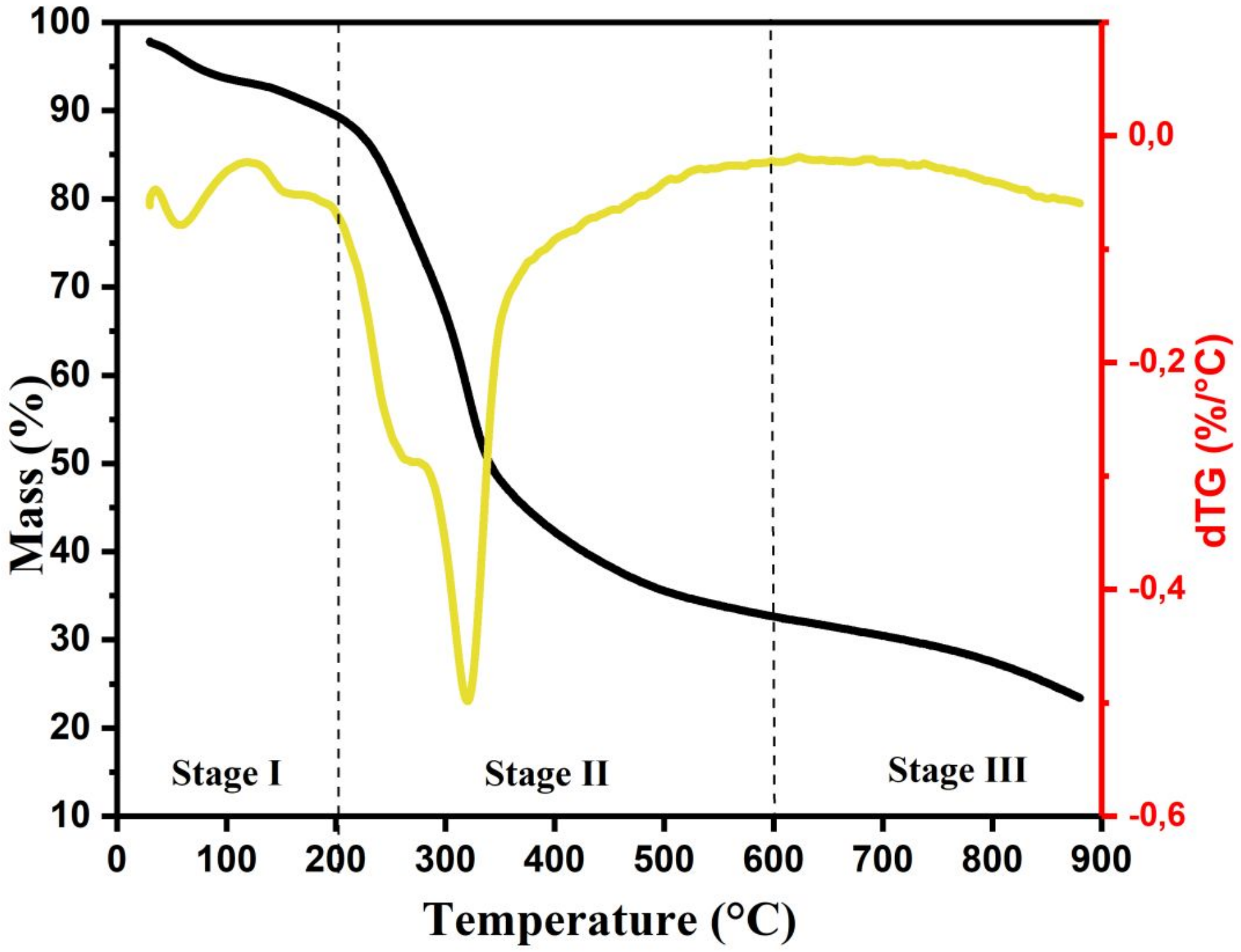

Fig.S3 TGA curve and first derivative obtained for biomass sample under N_2 atmosphere.

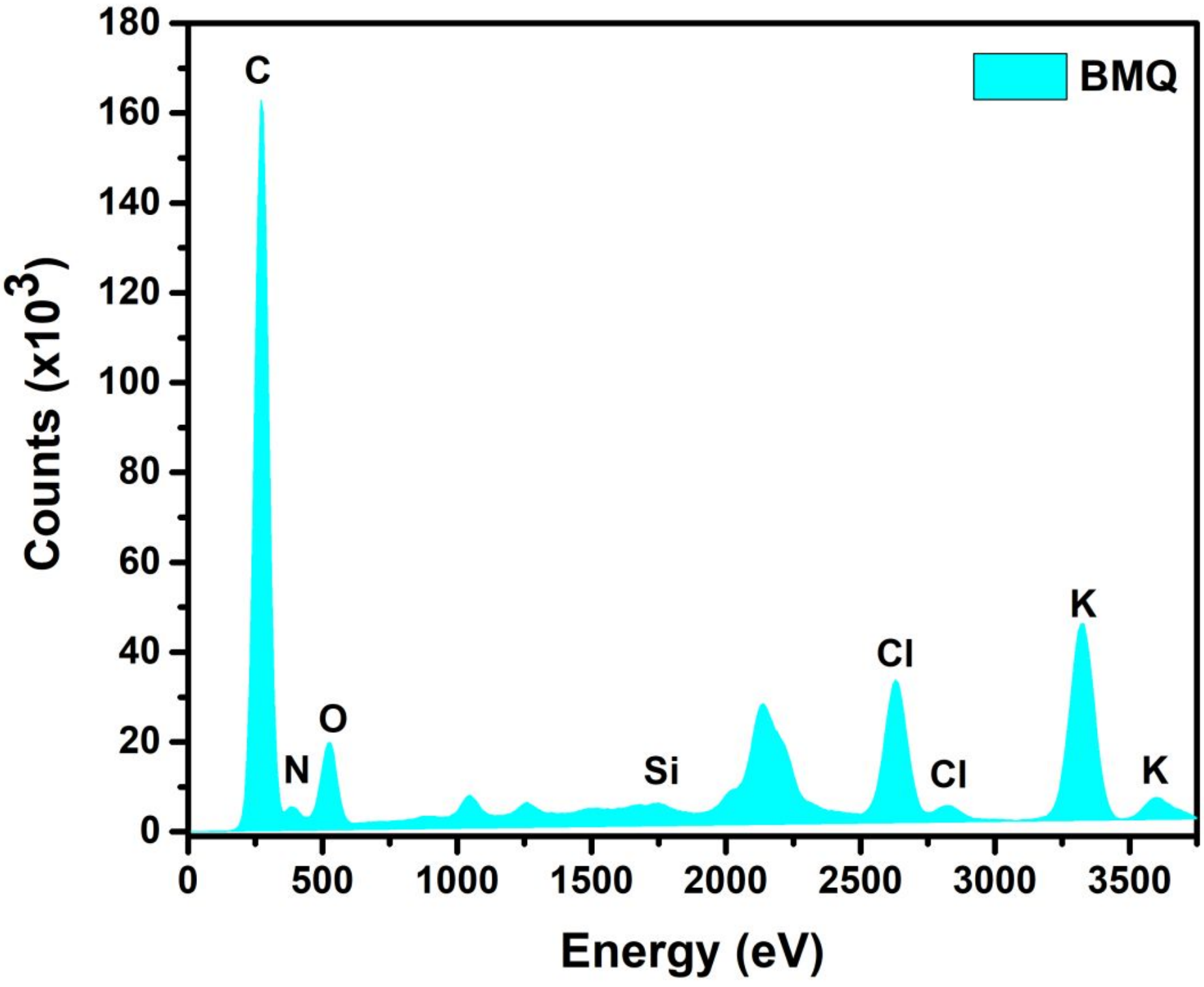

Fig. S4 EDS spectrum of the BMQ sample showing the elemental composition with signals of C, O, N, Si, Cl, and K

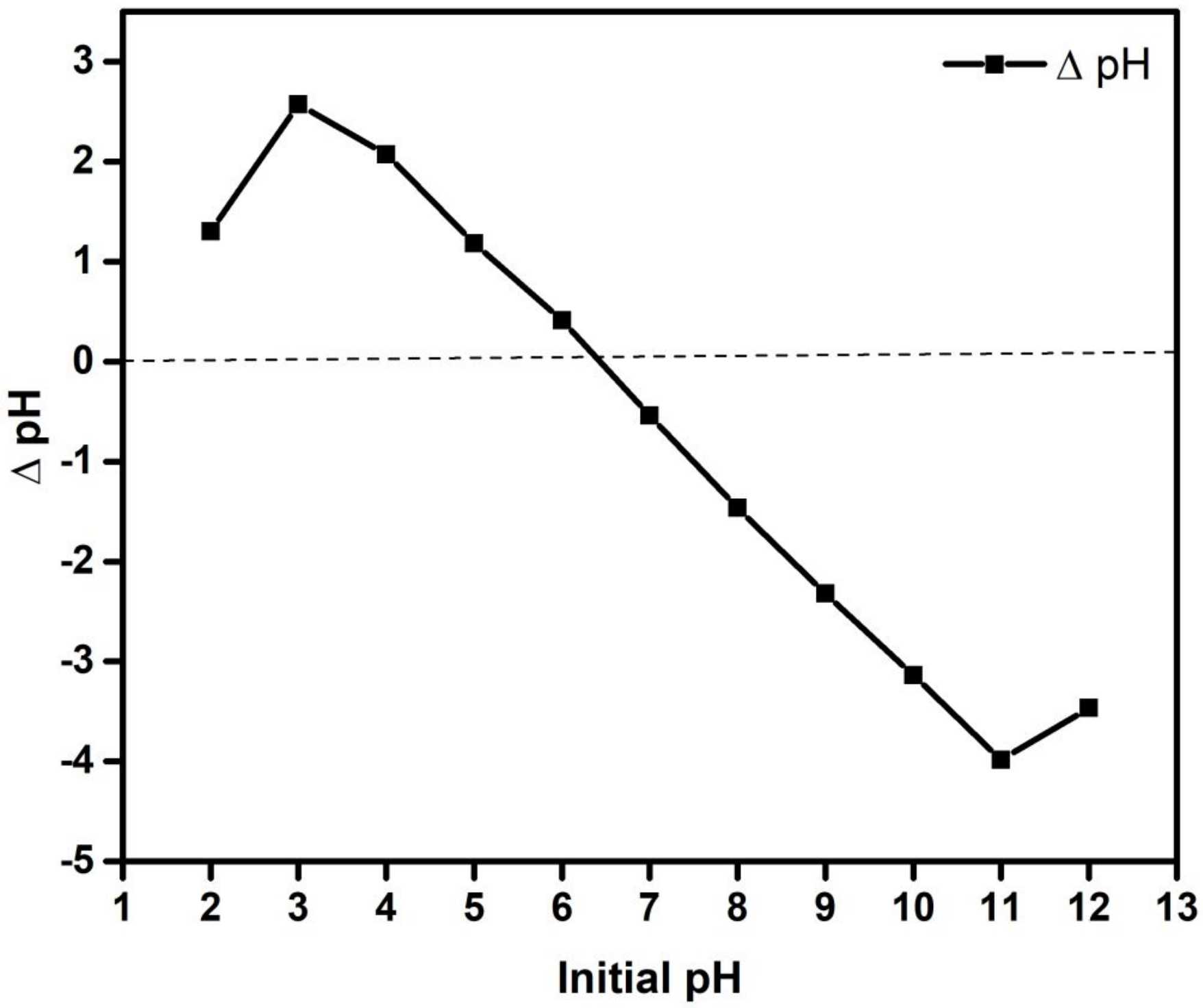

Figure S5 pH of initial solutions versus pH variations after contact of the BMQ sample for 24h.

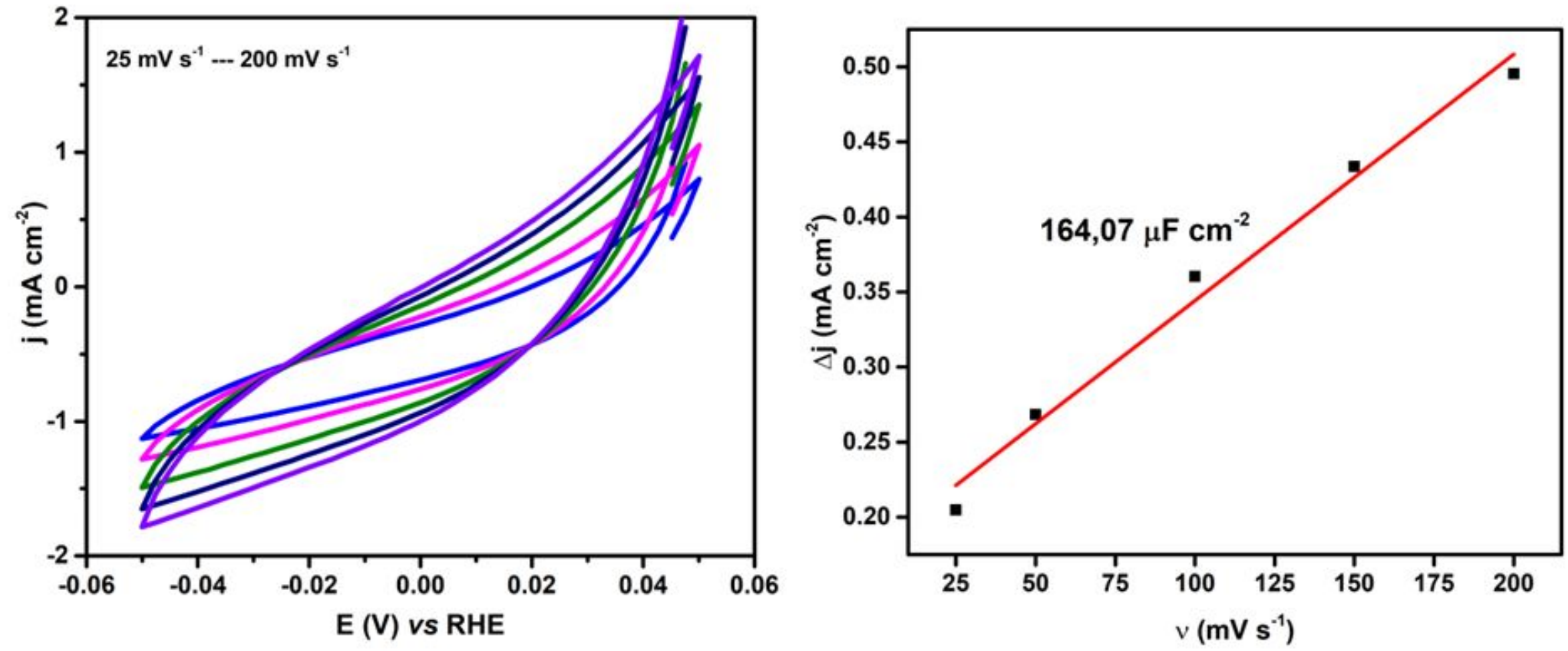

Fig. S6 Cyclic voltammetry curves of the BMQ sample recorded at different scan rates (25–200 mV·s⁻¹) in the non-faradaic potential region (left) and corresponding linear fit of Δj versus scan rate (right)

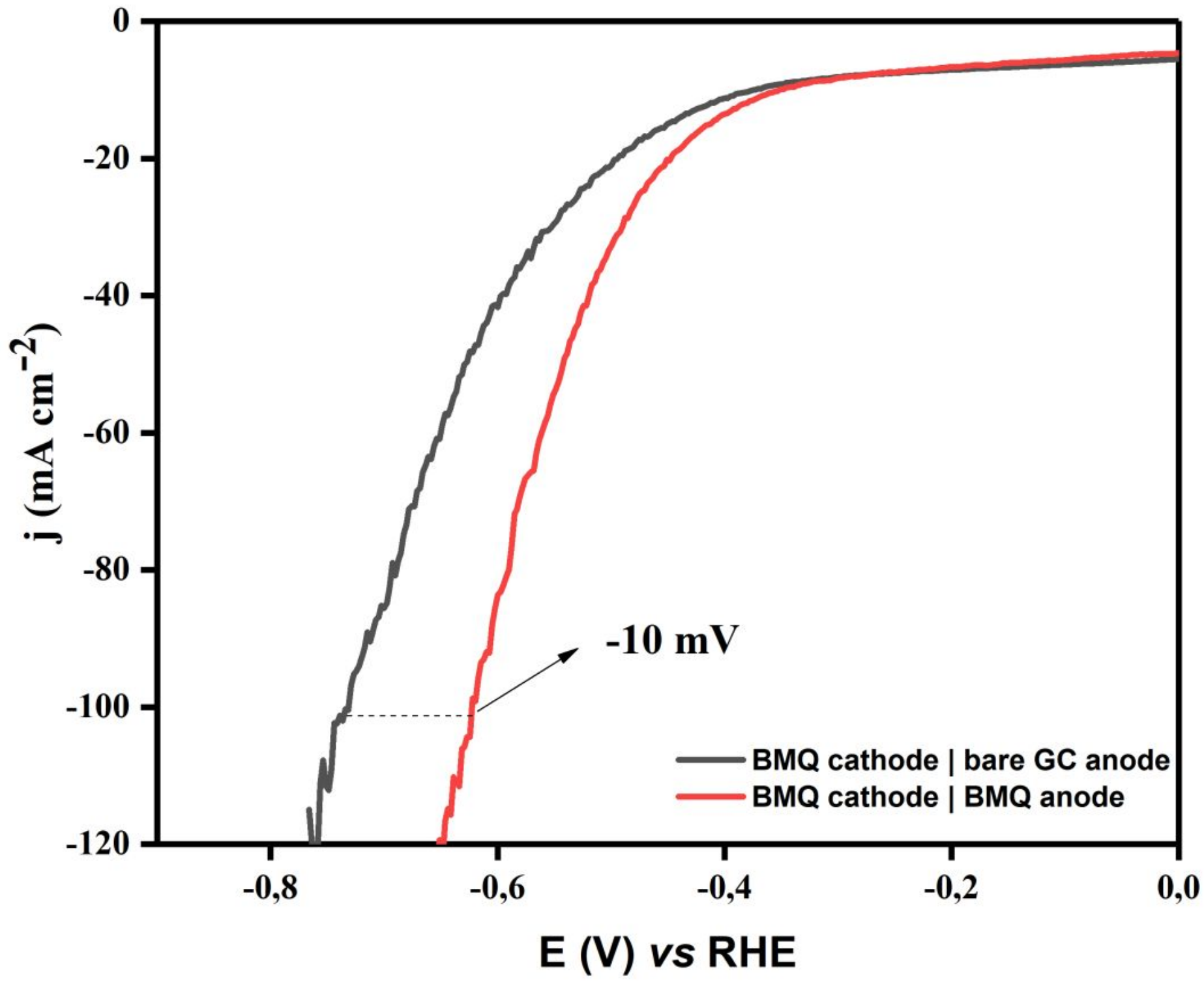

Fig. S7 Linear sweep voltammograms recorded for the HER using a BMQ-modified cathode coupled with either a bare glassy carbon anode (black curve) or a BMQ-modified anode (red curve).

TABLES

Region	Component	BE (eV)	FWHM (eV)	Line Shape	Relative Area (%)	Atomic Concentration (%)
**C1s**	C–C/C=C	284.50	1.28	GL(30)	33.74	38.78
**C1s**	C–O	285.55	1.50	GL(30)	44.86	36.37
**C1s**	C=O	286.93	1.36	GL(30)	13.94	16.72
**C1s**	O–C=O	288.80	1.25	GL(30)	7.46	7.14
**O1s**	C=O	531.33	2.30	GL(30)	61.88	54.31
**O1s**	C–O	533.00	2.44	GL(30)	38.12	45.69
**N1s**	Pyridinic-N	398.44	1.85	GL(30)	48.11	48.11
**N1s**	Pyrrolic-N	400.09	1.61	GL(30)	51.89	51.89

Table S1: Binding energies (BE), full width at half maximum (FWHM), line shapes, and relative contributions of the components used for XPS peak deconvolution of the BMQ sample.

HER				
**E (V)**	Rs (Ω)	Rct (kΩ)	Y0 (μS·s^n)	n
-0.30	0.95	7.21	39.3	0.740
-0.40	0.23	3.26	38.3	0.742
-0.50	2.89	2.72	53.2	0.696
-0.60	4.41	1.22	32.2	0.779
-0.70	5.34	1.07	30.3	0.788
**OER**				
+1.30	8.69	458	30.4	0.944
+1.40	7.27	212	24.4	0.936
+1.50	7.68	103	3.50	0.893
+1.60	4.42	51.8	20.0	0.686
+1.80	5.95	31.5	25.8	0.554

Table S2 Equivalent circuit fitting parameters obtained from EIS measurements at different potentials for HER and OER.

Element	Atomic %	Weight %
**Carbon**	84.18	75.81
**Oxygen**	8.13	9.75
**Nitrogen**	3.89	4.08
**Potassium**	2.14	6.26
**Chlorine**	1.23	3.28
**Silicon**	0.13	0.27

Table S3 Elemental composition of the BMQ sample obtained by EDS analysis, expressed in atomic and weight percentages.

Eletrocatalyst	η_{10} for HER (mV vs RHE)	Tafel slope for HER (mV dec^{-1})	η_{10} for OER (mV vs RHE)	Tafel slope for OER (mV dec^{-10})	Ref
**NaOH-ABC KOH–I ABC**<br>**KOH-II ABC**	231<br>213<br>188	168<br>146<br>137	155<br>126<br>90	146<br>133<br>127	1
**$Co_{0.15}$@ARC**	172	108	320	57	2
**BC-Mo/Co**	257	51	371	59	3
**NiFe@NCS-800**	284	102	371,5<br>(50 mV cm^{-2})	74	4
**C-450**	103	1.373	563	117	5
**$PrCoO_3$/ $Mn_2V_2O_7$@PBC**	121/ 71	123	282/ 582	36	6
**Ni-Mo_2C/NGC-800**	125	111	300	76	7
**Co@NC/NGC**	120	90	340	180	8
**RHB-CTP-CS/NF**	96.5	72.9	117.8	179.7	9
**Ru-CoNi@NLC**	50	40	170	**-**	10

**P-Ru/SC-2**	119.6 mV	67.5	193.4	125.2	[11]
**Co/N,S-CC**	0.46	107	570	89	[12]
**NiFeMo-P/CW**	70	52.17	230.2	85.22	[13]
**BMQ**	190	180	330	275	This work

Table S4: Comparison of the electrocatalytic performance of different electrocatalysts for the hydrogen evolution reaction (HER) and oxygen evolution reaction (OER).

REFERENCES

(1) Murugan, N.; Thangarasu, S.; Seo, S. Bin; Mariappan, A.; Choi, Y. R.; Oh, T. H.; Kim, Y. A. N-Doped Defect-Rich Porous Carbon Nanosheets Framework from Renewable Biomass as Efficient Metal-Free Bifunctional Electrocatalysts for HER and OER Application. *Renew. Energy* **2024**, *222*. https://doi.org/10.1016/j.renene.2023.119801.

(2) Zhou, Y.; Luo, Y.; Li, Q.; Liang, J.; Liu, F.; Cai, Y.; Lin, L.; Wu, Q.; Li, K. Cobalt Nanoparticles Encapsulated in the Biomass-Derived Carbon: An Efficient and Bifunctional Electrocatalyst for Overall Water Splitting. *Energy and Fuels* **2024**, *38*, 15560–15570. https://doi.org/10.1021/acs.energyfuels.4c01798.

(3) Ñanculeo, J.; Andreu, T.; Sirés, I.; Ramírez, A.; Cea, M.; Nahuelcura, B.; Valenzuela, G.; Garrido-Miranda, K.; González, M. E. Development of Hazelnut Shell-Derived Biochar to Support a Bifunctional MoCo Electrocatalyst for HER/OER in Alkaline Medium. *Biochar* **2025**, *7*. https://doi.org/10.1007/s42773-025-00464-0.

(4) Zhu, W.; Zhang, X.; Zhang, Q.; Chen, L.; Zhuang, X.; Ma, L. Biomass-Derived Catalysts with Dual Functions for Electrochemical Water Splitting. *Energies (Basel).* **2025**, *18*. https://doi.org/10.3390/en18143592.

(5) Kumaresan, N.; Karuppasamy, P.; Kumar, M. P.; Peera, S. G.; AlSalhi, M. S.; Devanesan, S.; Mangalaraja, R. V.; Ramasamy, P.; de Oliveira, T. F.; Murugadoss, G. Synthesis and Characterization of Metal-Free Nanosheets of Carbo-Catalysts for Bifunctional Electrocatalyst towards HER and OER Application. *Molecular Catalysis* **2023**, *539*. https://doi.org/10.1016/j.mcat.2023.113043.

(6) Aslam, S.; Safdar, M.; Mirza, M.; Abbas, Y.; Abbas, W.; Ali, B. Engineering Active Sites in Binary PrCoO3/Mn2V2O7 Heterointerface Embedded in Phosphorus-Doped Biochar for Boosting PH Universal Water Splitting. *Fuel* **2026**, *407*, 137409. https://doi.org/10.1016/J.FUEL.2025.137409.

(7) He, F.; Bai, Y.; Zhang, A.; Zhang, X.; Wang, F.; Wang, Q.; Zhong, M.; Su, B.; Dong, N. One-Step Synthesis of Ni-Mo2C/N-Biochar Heterojunction Bifunctional Electrocatalyst from Waste Grapefruit Peel for Efficient Overall Water Electrolysis. *Electronic Materials Letters* **2026**. https://doi.org/10.1007/s13391-026-00639-2.

(8) Zhong, M.; Liu, Y.; Chen, J.; Zhang, J.; Cao, J.; Li, W.; Su, B. N-Containing Biochar-Wrapped Co Nanoparticles Supported on N-Doped Graphitic Carbon for Efficient Alkaline Oxygen and Hydrogen Evolution Reactions. *Int. J. Hydrogen Energy* **2025**, *101*, 461–468. https://doi.org/10.1016/J.IJHYDENE.2024.12.488.

(9) Safarbeigi, H.; Khazalpour, S. Construction of a Nanocomposite Based on Rice Husk on Nickel Foam Substrate for Efficient Water Splitting in Alkaline Medium. *Energy Technology* **2026**, *14* (1). https://doi.org/10.1002/ENTE.202502333.

(10) Chen, D.; Liu, J.; Qiu, X.; Liu, B.; Wang, X.; Qiu, Z.; Lin, X.; Qin, Y. Lignin-Assisted Alloying Engineering of CoNiRu Trimetallic Nano-Catalyst for Effective Overall Water Splitting. *AIChE Journal* **2024**, *70* (3). https://doi.org/10.1002/AIC.18323.

(11) Li, P.; Cheng, Z.; Chai, W.; Yao, Y.; Zhang, N.; Yue, H.; Wang, Y.; Li, W.; Zhang, L.; Zhou, L.; Zhang, J.; Wang, Z.; Dong, T. Anchored Ru Clusters on a Lignin/Algae Carbon

Aerogel as an Efficient Bifunctional Catalyst for Water Splitting. *New Journal of Chemistry* **2024**, *48* (21), 9856–9861. https://doi.org/10.1039/D4NJ00409D.

(12) Matos, R.; Marques, I. S.; Fernandes, A. J. S.; Peixoto, A. F.; Fernandes, D. M. Multifunctional N,S-Doped Biochar Decorated with Transition Metals as Electrocatalysts for Energy Conversion Applications. *Biomass Bioenergy* **2026**, *204*. https://doi.org/10.1016/j.biombioe.2025.108463.

(13) Xu, Q.; Hua, J.; Wang, Y.; Cheng, G. Beechwood-Derived NiFeMo-P/CW Hierarchically Porous Bifunctional Electrocatalysts for Efficient Water Splitting. *J. Power Sources* **2025**, *635*. https://doi.org/10.1016/j.jpowsour.2025.236471.
